# Numerical assessment of arterial punch method and arterial reconstruction in femoral endarterectomy

**DOI:** 10.1007/s10439-025-03776-1

**Published:** 2025-06-24

**Authors:** Dongxu Liu, David Jiang, Efi Efrati, Nhung Nguyen, Luka Pocivavsek

**Affiliations:** 1 Department of Surgery, The University of Chicago, Chicago, USA; 2 Department of Physics of Complex Systems, Weizmann Institute of Science, Rehovot, Israel

**Keywords:** Arterial punch, Arterial reconstruction, Finite element methods, Artery restenosis, Femoral endarterectomy

## Abstract

**Purpose:**

Endarterectomy, typically in patients with peripheral artery disease, involves arteriotomy closure with a ‘patch.’ One of its most common long-term complications is restenosis due to arterial wall hyperplasia induced by excessive mechanical stimulation. Methods to reduce surgically induced stress to promote positive long-term outcomes remain an open question.

**Methods:**

In this work, an arterial ‘punch’ approach is proposed to alleviate the stress concentration in arterial walls around the incision/patch anastomotic interface. Intraoperatively, coronary vascular punches are used to create proximal and distal circular arteriotomies for patients undergoing femoral endarterectomy. The surgical procedure is numerically simulated by first opening the vessel wall and subsequently adjusting the boundary condition of the incision to consider the patch’s effect. An optimization study is performed by investigating the impact of incision/patch combinations on arterial wall stresses. The optimal punch tip size is identified by obtaining the minimum in-plane principal stress in the arterial wall. A beveled punched hole is also considered to optimize the stress field.

**Results:**

Simulation results show that the stress magnitude in the arterial wall with a punched hole is significantly lower than that in an artery with a sharp vertex. The stress exponentially declines with increasing punch diameter. A beveled hole can further reduce the stress values and the number of high-stress regions.

**Conclusion:**

The arterial punch method can effectively alleviate stress concentration in arterial tissues. Importantly, stress concentration is shown to be sensitive to punched hole size and shape, suggesting potential practical implications for surgical techniques and patient outcomes.

## Introduction

Peripheral artery disease is an atherosclerotic disease process that impacts 7% of the population of the United States [1]. The development of clinically meaningful stenoses is multifactorial and includes patient and biomechanical factors. Clinical risk factors include concomitant diseases such as diabetes mellitus and hypertension, biological elements such as circulating lipoprotein levels, and genetic contributions [2]. Mechanical risk factors include native arterial morphology and regions of concentrated wall stress. Surgical removal of symptomatic atherosclerotic plaque is often necessary to restore adequate blood flow to the extremities, and this is accomplished by a femoral artery endarterectomy. An arteriotomy, or incision, is created in this operation over the common and profunda femoris arteries with manual removal of plaque. Closure of the arteriotomy, patch angioplasty, is accomplished with a xenograft, such as those derived from bovine pericardium [3, 4]. Closure with a patch introduces an incision/patch anastomotic interface.

Arterial walls have fundamentally complex biological structures composed of elastin, collagen, smooth muscle cells, and endothelium [5]. These biological components are sensitive to physiologic loading. Subject to normal dilation/contraction force from diastolic/systolic pressure, they respond to mechanobiological stimulation and achieve a homeostatic state. However, perturbations of the arterial wall are common in disease states such as atherosclerosis and in iatrogenic cases of vascular surgery, where tissue homeostasis is interrupted due to abnormal mechanical stresses [6]. The response of these tissues to disease and surgery has been thoroughly investigated. For example, chronic hypertension could significantly decrease the half-life of collagen [7]. Olivetti et al. observed smooth muscle cell volume increase due to aortic-coarctation-induced hypertension [8]. Vessel restenosis and optimal healing are complex with multiscalar components [9]. Thus, additional insight is required to develop techniques to mitigate this negative outcome.

A traditional femoral arteriotomy involves the creation of a linear incision on the anterior surface of the femoral arteries. The incision has acutely angled V-shaped notches at its vertices [10]. To ensure a minimal removal of arterial wall tissues, the gap in the incision of undeformed arterial walls is narrow [5, 11]. An analogous methodology of understanding these shapes is seen in the study of conical defects in differential geometry. The resultant incision/patch system results in a conical geometry due to the geometric mismatch between the incision and patch; thus, a working hypothesis in this study proposes that the pressurized anastomosis can be studied as a relatively thin sheet with ‘*excess cone*,’ i.e., *e-cone*, generated at its apical vertices with remote ‘*developable cone*,’ i.e., *d-cone*, perturbations, both of which induce non-native stress focusing, due to the patch angioplasty being a material-adding process [12–16]. This incision/patch geometric mismatch subsequently increases the risk of neointimal hyperplasia and restenosis [17–19]. Furthermore, the potential compliance mismatch due to the different mechanical properties between the patch and arterial wall can be another source for elevated stress concentrations [10, 20]. In contrast to the sharp-end incision in femoral arteriotomy, rounded arteriotomies are particularly common in coronary artery bypass graft operations, where vascular punches have long been used for aortotomies before aortosaphenous vein end-to-side anastomoses [21, 22]. The vascular punch is preferred in this situation because it creates circular and symmetrical arteriotomies through the aortic wall. The circular shape can effectively alleviate stress singularity at the incision vertices, motivating an alternative arterial reconstruction strategy in femoral endarterectomy.

Although the V-shaped and round-shaped incision/patch anastomoses were modeled to study the tissue biomechanical behavior in artery patch reconstruction in previous numerical studies [5, 10, 23], to date, no rigorous study has analyzed how differing conical geometries at the vertices of these anastomoses impact the mechanical stresses that develop and, subsequently, the biological response to the altered geometry and stress concentration. In the present work, we propose a computational method to systematically analyze the effect of geometries at the incision/patch anastomotic site on the biomechanical response of artery tissues. Three anastomotic methods are modeled and compared to find the optimal geometric match between the incision and patch. The relationship between punch sizes and stress concentration factors is quantified. Simulation results demonstrate that a punched hole significantly reduces stress concentration; increasing this hole diameter further mitigates non-homeostatic stressors; a beveled punched hole is the optimal arteriotomy apex geometry to optimize stress distributions. The simulation results can provide theoretical support for developing new endarterectomy surgical strategies to reduce restenosis risk.

## Materials and Methods

### Operative Details

Informed consent was obtained from the patient for research purposes. Institutional review board approval was waived.

A 66-year-old patient was initially diagnosed with peripheral arterial disease (PAD) several years prior to this operation. He subsequently underwent bilateral below-knee amputations and was able to ambulate using a prosthesis until 6 months before presentation, when he developed a chronic, poorly healing left lower extremity wound. Pre-operative duplex ultrasonography demonstrated a peak systolic velocity (PSV) increase from 62 to 350 cm/s at the left common femoral artery and a PSV increase of 72–218 cm/s at the left mid-superficial femoral artery, consistent with hemodynamically significant stenosis. Diffuse calcific plaques were also noted, which were consistent with computed tomography angiography (CTA) imaging. CTA demonstrated multifocal left lower extremity stenosis that was most severe at the common femoral and popliteal arteries. The left profunda femoris artery was densely calcified, and the left superficial femoral artery was partially occluded.

**Fig. 1:**
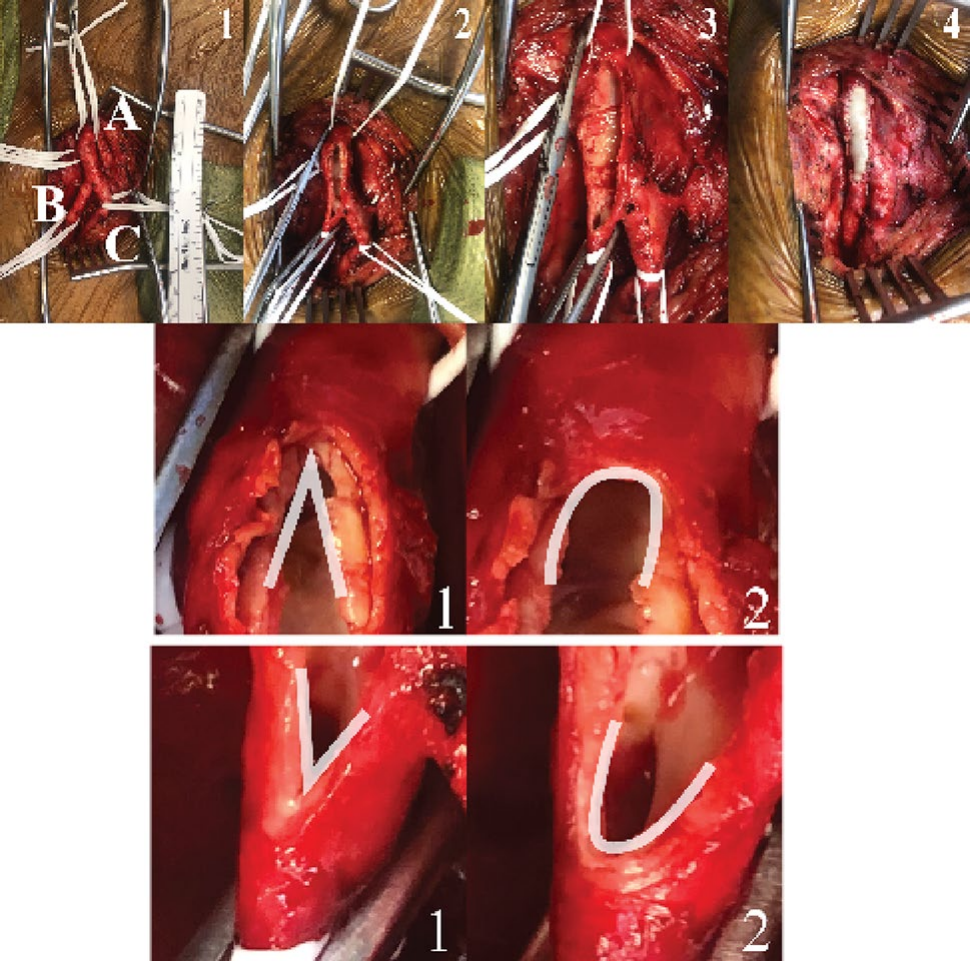
Upper panel illustrates the operative procedure of femoral endarterectomy with patch angioplasty. Upper Panel 1 represents an exposure of the femoral arteries with labels corresponding to the common femoral artery (**A**), profundal femoris artery (**B**), and superficial femoral artery (**C**). Upper Panels 2 and 3 demonstrate the creation of an arteriotomy and subsequent use of a vascular punch to generate beveled apices, followed by patch suturing in Panel 4. The middle and lower panels show a detailed view of the common femoral artery (Middle Panel 1 and 2) before and after beveling, and the profundal femoris artery (Lower Panel 1 and 2) before and after beveling using a vascular punch. The white outline in the middle and lower panels highlights the region of interest, with a significant reduction in the acuity of the angle at the vertices of the vessels after beveling

The patient provided informed consent and underwent a left femoral endarterectomy with patch profundoplasty. The operation began with infrainguinal exposure of the left common femoral, superficial femoral, and profunda femoris arteries. A linear arteriotomy was created on the anterior surface of the common femoral artery and extended onto the profunda femoris artery. An endarterectomy was performed using the freer elevator. The distal external iliac plaque was removed using an eversion endarterectomy technique. The vertices of the common femoral artery and the profunda femoris were then rounded using a 4.0-mm coronary punch, as shown in Fig. 1. A bovine pericardial patch profundoplasty was then performed by suturing the patch to the arteriotomy. These commonly used patches have diamond-shaped tips (see Fig. 3d) with an angle of around 45°. It is hypothesized that rounding the tip of the linear incision decreases the stress at the cut vertices.

### Numerical Simulation

In this section, the mechanical responses of arteries, reconstructed with different patching strategies, are simulated using Finite Element Methods (FEM). The effect of geometric mismatch between incisions and vascular patches on the biomechanical response of arterial walls is simulated and analyzed systematically. We first investigate the impact of incision and patch shapes on the mechanical performance of reconstructed arteries, followed by determining the optimal punch size. Finally, a beveled punched hole is introduced to further optimize the stress distribution surrounding the punched hole. The simulations are performed using the commercial finite element software Abaqus/Explicit (Version 2021, Dassault Systèmes Simulia Corp., Johnston, RI, USA). The computations were conducted on a computer equipped with 12-cores 12th Gen Intel(R) Core(TM) CPU i7-12700 (base speed 2.10 GHz, 32 GB RAM). On average, each simulation requires approximately 10 h. All plots and fitting are performed using the software OriginPro (Version 2018, OriginLab Corp., Northampton, MA, USA).

#### Geometry and Boundary Conditions

**Fig. 2:**
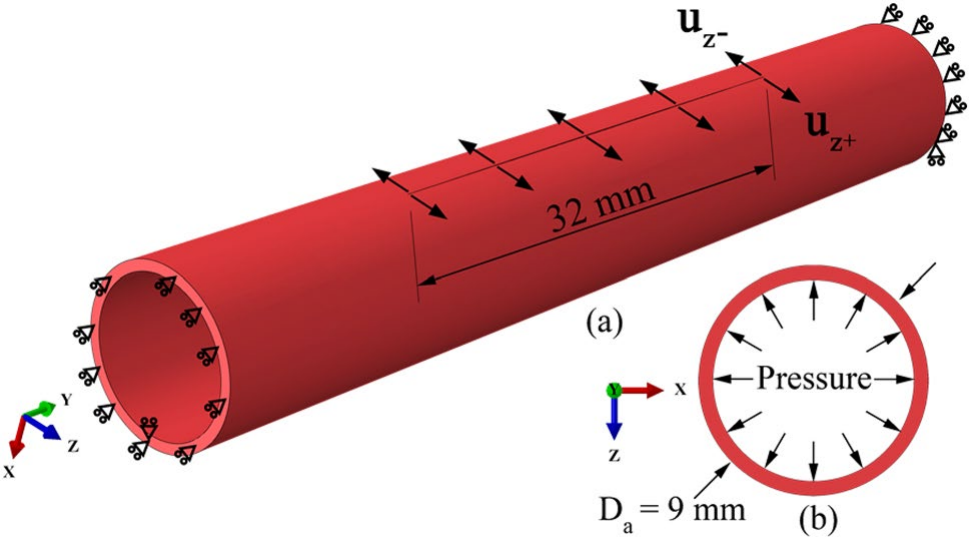
**a** Geometry and boundary conditions, and **b** loading conditions (pressure) of the computational models

**Fig. 3:**
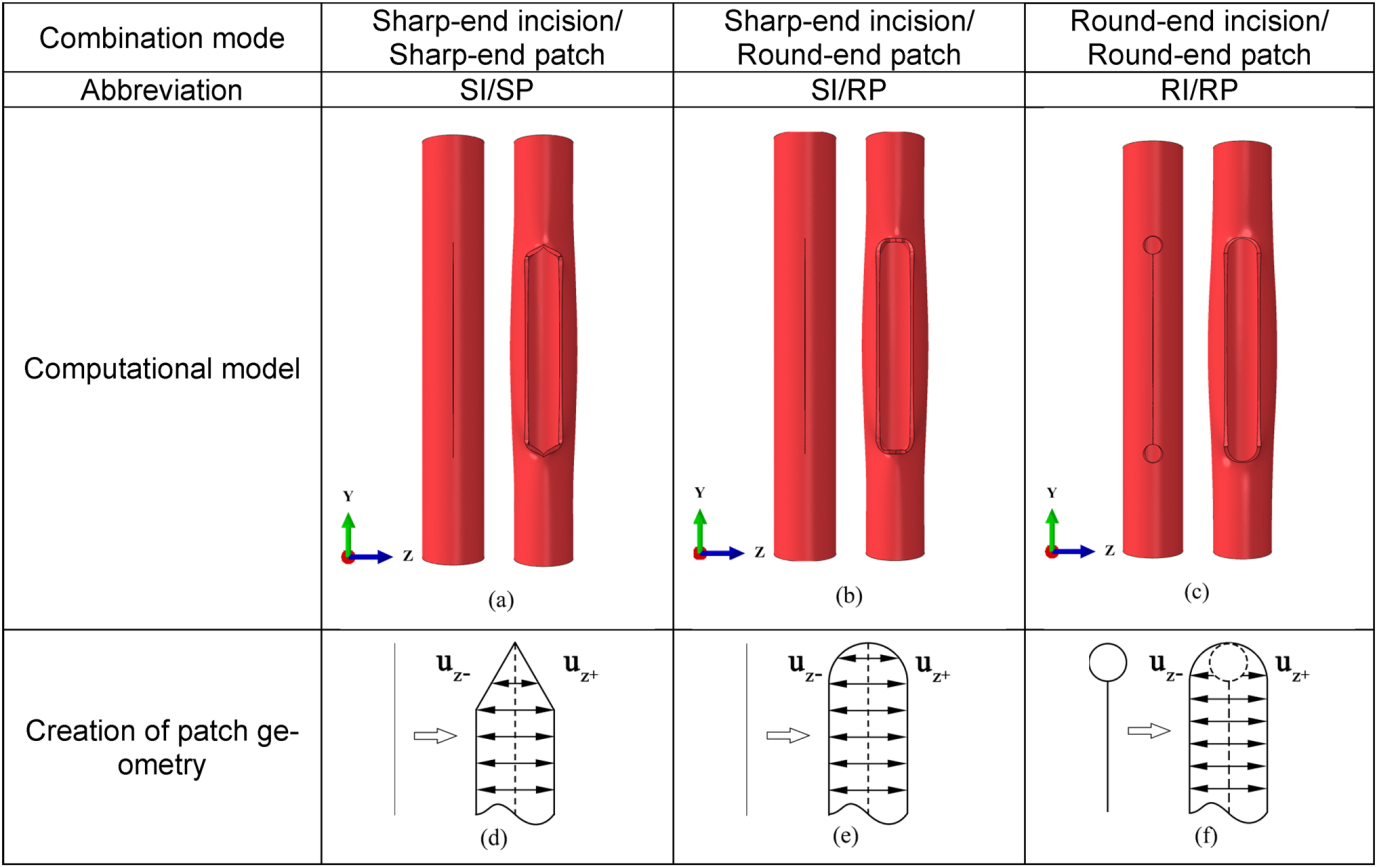
The geometry of models with different combination modes between incisions and patches: **a** SI/SP; **b** SI/RP; **c** RI/RP. For simplicity, the abbreviations of the combination modes are used in the following sections. In the RP cases, the diameter of the punched hole is 2.8 mm, which was used in surgeries, see Fig. 4a. The schematics of the method for patch geometry creation in a–c are illustrated in **d**–**f**, respectively. The diameter of the round end of the patch is 5 mm

The geometry, dimensions, and boundary conditions of the computational models are shown in Fig. 2. Arterial models are simplified as cylinders according to native human femoral arterial dimensions, as shown in Fig. 1. The dimensions of the computational models are approximated from patient data. The longitudinal length of the modeled artery is 64 mm, and the diameter is 9 mm [24]. The thickness of the artery is assumed to be 0.6 mm [25]. A 32-mm incision is created at the center of the anterior surface to represent the arteriotomy. In simulations, two consecutive analysis steps are created to simulate the surgical procedure. In step 1, the incision is opened by moving the incision edges in Z + and Z− directions to match the boundary of the desired patch (Figs. 2a and 3d–f). The movement of the cross-sectional surfaces in the Y direction is restricted to consider the constraint of the connected artery. Two points on the cross-sectional surfaces are fixed in the X direction to avoid spatial translation. Compared with arterial wall tissues, the material of some typical patches, for example, polytetrafluoroethylene (PTFE), Dacron, and bovine pericardium, is stiffer [10]. Since the patch is stiffer than the arterial wall and is assumed to be rigid, its stretches in the Y (longitudinal) and Z (width) directions are negligible. In addition, the movement of the patch in the X (radial) direction is assumed to be small and negligible. Therefore, the movements of the incision/patch border in all directions are fixed during step 2 of the simulations. The strengths of this assumption include (1) the procedure of building the complex nonlinear geometry of the computational model is simple; (2) the computational cost is reduced. A 10-kPa pressure, a representative of a standard mean arterial pressure for non-pulsatile loading [26], is uniformly applied to the internal surface of the artery and linearly increases in step 2 (Fig. 2b).

##### Comparison Between Different Arteriotomy and Reconstruction Strategies

In angioplasty, a patch is sutured to the incision to reconstruct the artery. The patch is cut with tips at both ends to match the incision geometry. We first compare three different tip configurations after angioplasty, as shown in Fig. 3, i.e., sharp-end incision/sharp-end patch (SI/SP), sharp-end incision/round-end patch (SI/RP), and round-end incision/round-end patch (RI/RP).

##### Determination of Punch Sizes

**Fig. 4:**
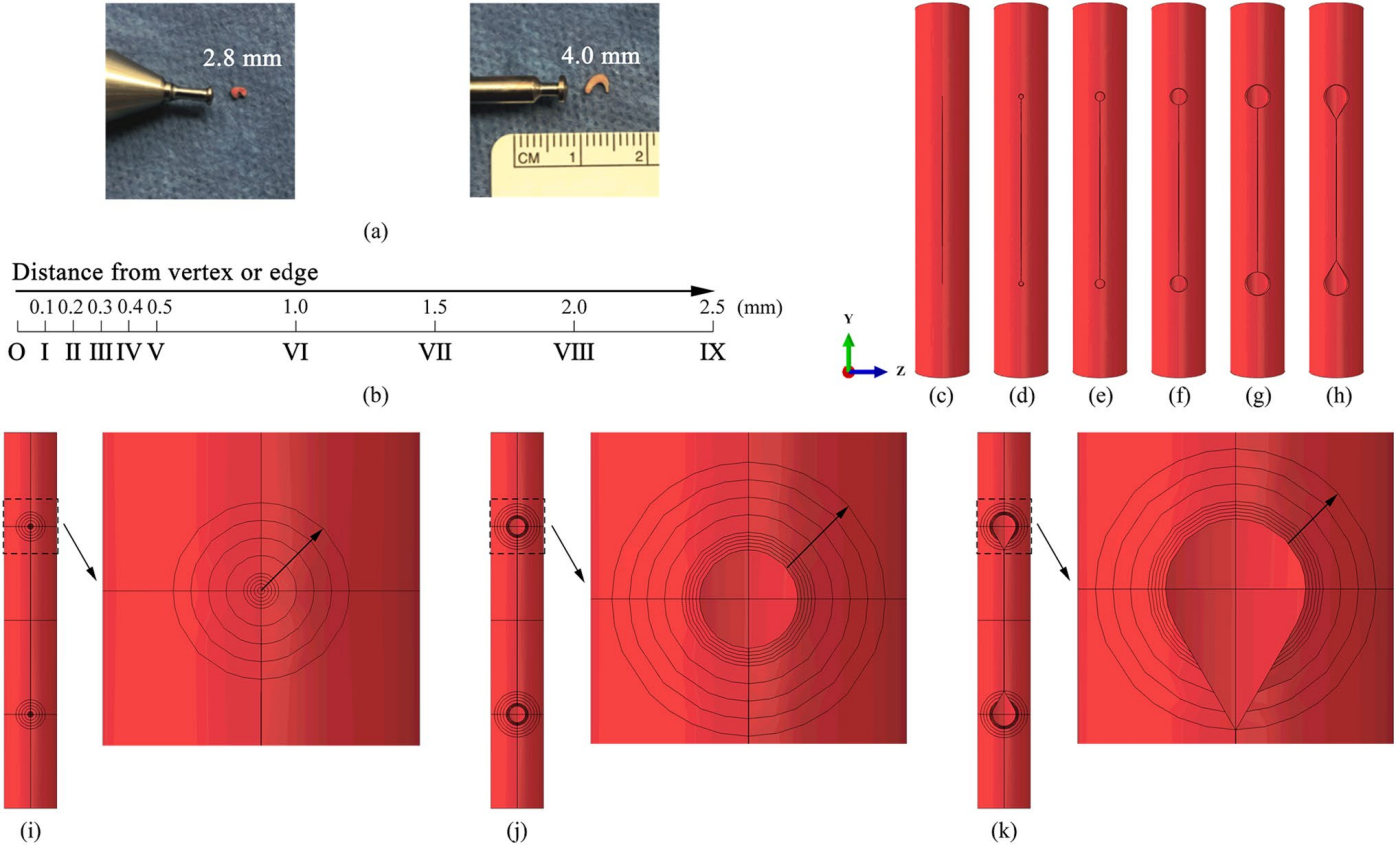
**a** A 2.8-mm and a 4.0-mm vascular punch are used to remove tissues around the tips of incisions; Zone I to Zone IX in **b** correspond to the nine circular zones in (**i**) and (**j**). Only eight zones (Zone I to Zone VIII) are defined in the 4.0-mm RI model and BRI model due to the limitation of arterial diameter (**k**); Sizes of the punched holes: **c** 0 mm (SI); **d** 0.8 mm; **e** 1.6 mm; **f** 2.8 mm; **g** 4.0 mm. A beveled hole **h** is created based on the 4.0-mm punched hole; Definition of zones for average stress calculation in (**i**) SI model, (**j**) 2.8-mm RI model, and (**k**) beveled round-end incision (BRI) model

Four sizes are selected to quantify the influence of punch diameter on the stress magnitude and distribution. A 2.8- and a 4.0-mm punches are used in surgeries (Fig. 4a). Another two diameters, i.e., 0.8 and 1.6 mm, are selected in simulations for comparison. The models with different sizes of punched holes are shown in Fig. 4c–h.

##### Optimization of the Punched Hole Geometry

Punching the incision vertices creates circular holes. It concomitantly introduces two corners at the connection between the hole and the remaining linear incision, see Fig. 4d–g, which could redistribute the stress surrounding the holes. An alternative method is creating a beveled hole by removing the tissues at the connection regions. This method was carried out in surgeries, see Fig. 1. To demonstrate the effectiveness of the beveled hole, a model with beveled punched holes is created based on the 4.0-mm RI model, as shown in Fig. 4h.

The meshes of the computational models at the regions surrounding both ends of the incision are refined by S3R elements with a minimum element size of 0.1 mm to better resolve the stress field. The element size is determined by conducting a mesh convergence analysis, as shown in Appendix A, where no significant difference in stress magnitude and distribution between models with different element sizes is observed. The remaining region is meshed by S4R elements with a mesh size of 0.25 mm.

#### Material Properties

The femoral artery is modeled as a Neo-Hookean hyperelastic material [27, 28]. The mass density of the artery wall is assumed to be 1.12 × 10^−9^ g/mm^3^ [29]. The initial shear and bulk moduli are assumed to be 0.0604 and 0.333 MPa, respectively [5, 10].

#### Evaluation of Average Stress

The average stress of the tip region is calculated to analyze the effect of tip geometry on stress distributions. The stress surrounding the vertices of the incision is averaged over predefined circular zones. The distance from the vertex is used as the radius of the circular zones in the SI model, and the distance from the edge of the punched hole is used to determine the size of the circular zones in the RI models (Fig. 4i–k). The distance ranges from 0.1 to 2.5 mm (Fig. 4b).

#### Calculation of Stress Concentration Factor

Stress concentration factor *K* is calculated to characterize and quantify the degree of stress concentration in models with different repair approaches. In the simplest system of an infinite plane under tension with an oval or circular hole in plane, the factor is calculated as ${K}_{t}=1+2\sqrt{a/\rho }$, where $a$ is the width of the hole and $\rho$ is the root radius. Small values of $\rho$ yield high ${K}_{t}$. For the arterial incision, we expect that larger root radii reduce the stress concentration factor. We apply a similar dimensionless ratio to this Neo-Hookean hyperelastic system to quantify differences in stress concentration among different geometries. The stress concentration factor *K* is defined as the ratio of the maximum stress (${\sigma }_{max}$) at the vertex or punched hole edge to the reference stress (${\sigma }_{ref}$), i.e., $K={\sigma }_{max}/{\sigma }_{ref}$. The far-field stress is adopted as the reference stress.

## Results

### Clinical Outcome

The patient was evaluated in the clinic one month post-operatively when he was recovering appropriately with the general improvement of his symptoms. The previously chronic wound of the left lower extremity demonstrated signs of healing with new granulation tissue in the wound bed and no clinical signs of infection. Arterial duplex ultrasonography was performed; the left femoral endarterectomy site was patent. PSV at the common femoral artery was substantially lower at 68 cm/s compared with 350 cm/s pre-operatively.

### Finite Element Analysis

The variation of stress in arterial walls is mainly caused by the incision-opening-induced change of geometry and pressurization. The simulations are correspondingly divided into two steps. In step 1, the incision is opened by modulating the displacement of the edges of the incision to a specified width. The final shape of the opened incision at the end of this step matches the shape of the patch to simulate the patched arterial wall. In step 2, non-pulsatile mean arterial pressure is applied to the intima incrementally to simulate the effect of pressurization on a completed repair.

#### Comparison Between Different Arteriotomy and Reconstruction Strategies

**Fig. 5:**
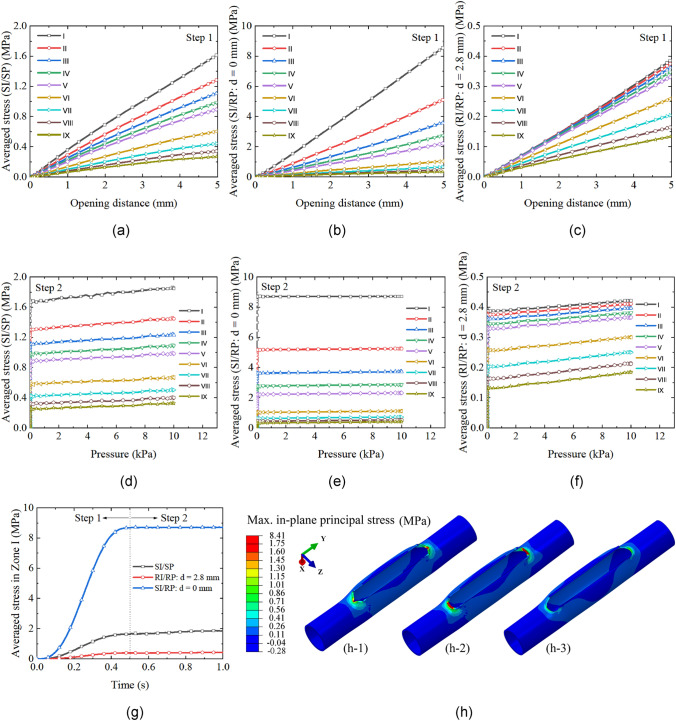
Evolution of averaged stress with opening distance (**a**), (**b**), (**c**) and pressure (**d**), (**e**), (**f**) in the reconstructed artery with SI/SP, SI/RP, and 2.8-mm RI/RP, respectively. The opening distance is defined as the distance between the two parallel edges of the incision, as shown in Fig. 3; **g** Comparison of the stress evolution in Zone I among the reconstructed artery models with three methods; **h** Comparison of the stress distribution among the reconstructed artery models with three methods: (**h-1**) SI/SP; (**h-2**) SI/RP; (**h-3**) 2.8-mm RI/RP

The performance of the considered punch and patch strategies, as shown in Fig. 3, is first compared. Using the proposed method in “Evaluation of Average Stress” Sect., the evolution of averaged stress with opening distance and pressure in the reconstructed arteries with SI/SP is calculated and presented in Fig. 5a and d. The averaged stresses in all zones increase with the opening of the incision in step 1, while stresses increase slightly in the pressurization step. The stress is the highest in Zone I and declines with the extension of the zone area.

The calculated averaged stresses in the artery with SI/RP are demonstrated in Fig. 5b and e. The overall trend of stress evolution in all zones is the same as that in the artery with SI/SP. However, the stress magnitude is significantly higher. The difference is more apparent in Zone I to Zone V.

The stress in the artery reconstructed with RI/RP is shown in Fig. 5c and f. It is worth noting that the averaged stresses in Zone I to Zone V roughly overlap. The stress in this case is the lowest among the models with three different artery reconstruction strategies that are demonstrated in Fig. 3. For better quantifying their difference, the comparison of the stresses in Zone I is shown in Fig. 5g.

The stress distributions in the three scenarios are presented in Fig. 5h. The stress in the SI/RP model concentrates in Zone I. In the SI/SP model, the stress also concentrates in Zone I, but it slightly spreads out and distributes in the tissues on the shoulder of the patch. The maximum stress in the RI/RP model is the lowest compared with the other two, and the stress in the arterial walls is distributed in an obviously larger region.

#### Determination of Punch Sizes

According to the results in “Comparison Between Different Arteriotomy and Reconstruction Strategies” Sect., punching the sharp corners of an incision to create circular holes is an effective solution for alleviating stress concentration. In this section, the influence of the punch size is investigated, and the optimal punch size is determined. Four punch diameters are considered, namely 0.8, 1.6, 2.8, and 4.0 mm, as shown in Fig. 4d–g. The sizes are selected based on the punch sizes used in surgeries (Fig. 1).

**Fig. 6:**
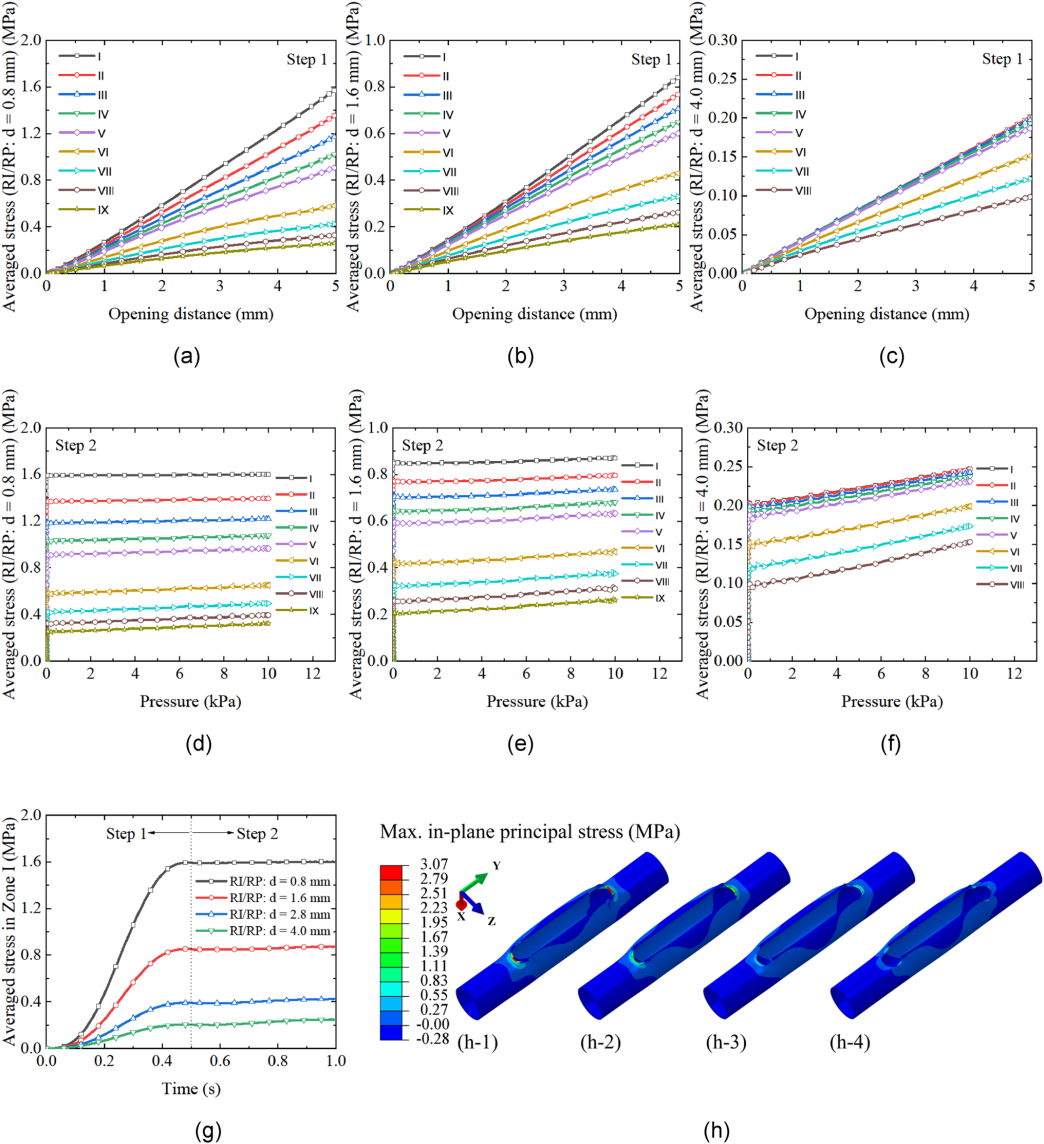
Evolution of averaged stress with opening distance (**a**), (**b**), (**c**) and pressure (**d**), (**e**), (**f**) in the reconstructed artery with 0.8-, 1.6-, and 4.0-mm RI/RP, respectively; **g** Comparison of the stress evolution in Zone I among the reconstructed artery models with four punch sizes; **h** Comparison of the stress distribution among the reconstructed artery models with four punch sizes: (**h-1**) 0.8-mm RI/RP; (**h-2**) 1.6-mm RI/RP; (**h-3**) 2.8-mm RI/RP; (**h-4**) 4.0-mm RI/RP

The stress-opening distance relationships in step 1 and stress–pressure curves in step 2 obtained from the models with the four punch sizes are plotted in Figs. 5c and f and 6a–f. The averaged stresses in all zones drop with the rise of punch size. The stress within Zone I is the highest in the model with 0.8- and 1.6-mm punched holes, and the stress declines with the growth of the circular zone diameter. However, there is a switch when the punch size increases to 2.8 mm, larger than which the difference of stress in Zone I to Zone V is not significant. In the 4.0-mm RI/RP model, it is noted that the stresses in Zone I to III almost overlap and are the highest among all circular zones in this model. This trend is different from the other three cases, where the averaged stress changes monotonically with the expansion of zone areas.

To compare the variation of stress caused by the change of punch size, the averaged stresses calculated within Zone I for the models with different punch sizes are illustrated in Fig. 6g. Stresses increase due to the opening of the incision in step 1, while they remain nearly constant in step 2, where a rigid patch is assumed to be sutured to reconstruct the artery. Averaged stress within Zone I decreases when a larger punch size is used to remove the tissue at both ends of the incision. The stress distributions in the whole computational domain are shown in Fig. 6h. It is obvious that the maximum stress decreases, and the high-stress zone expands to a larger region with the usage of a larger punch size.

#### Optimization of the Punched Hole Shape

**Fig. 7:**
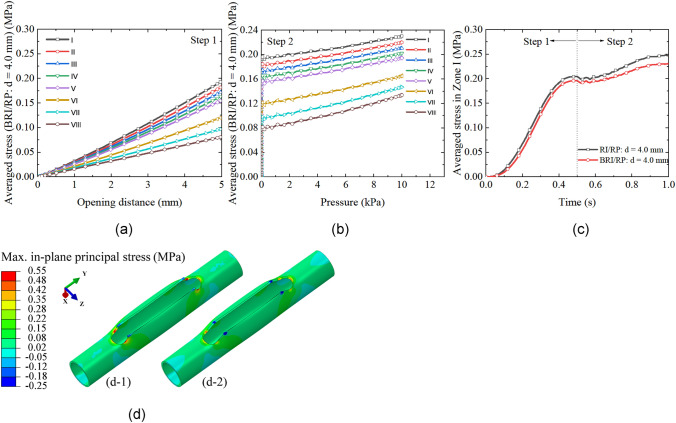
Evolution of averaged stress with (**a**) opening distance and (**b**) pressure in the reconstructed artery with 4.0-mm BRI/RP; **c** Comparison of the stress evolution in Zone I between the reconstructed artery models with 4.0-mm RI/RP and 4.0-mm BRI/RP; **d** Comparison of the stress distribution between the reconstructed artery models with (**d-1**) 4.0-mm RI/RP and (**d-2**) 4.0-mm BRI/RP

Although a reasonably large punch size can effectively distribute the stress surrounding the incision corners, a large hole introduces other high-stress zones away from the vertex region. As illustrated in Fig. 7d-1, stress concentrates at the top of the incision and both shoulders. A beveled hole is created based on the 4.0-mm punched hole by removing the tissues at the connection region between the hole and the linear incision, which is depicted in Fig. 4h. An approximated arithmetic descent of averaged stresses is observed with the increase of circular zone area, see Fig. 7a and b. The comparison of averaged stress within Zone I between the model with standard 4.0-mm punched holes and the model with a beveled hole is shown in Fig. 7c. The introduction of the beveled hole further lowers the stress levels in Zone I. The corresponding overall stress distribution in the beveled-hole model is shown in Fig. 7d-2. The stress levels at the hole region are obviously alleviated, compared with the stresses at the same regions in the circular punched hole model (Fig. 7d).

### Characterization of Stress Concentration

**Fig. 8:**
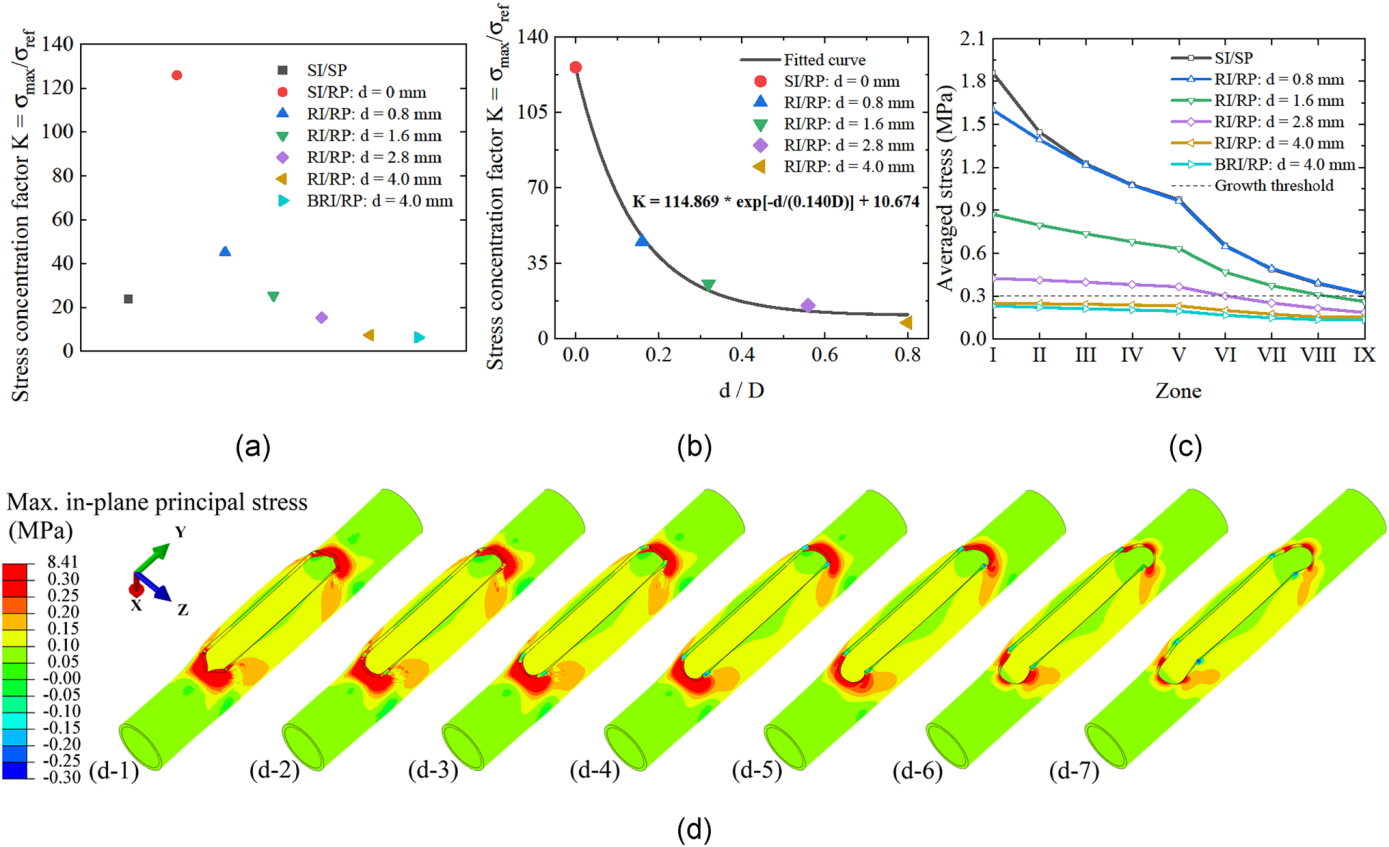
**a** Stress concentration factor $K$ for all cases; **b** Fitting curve and stress concentration factor $K$ for models with different diameters of punched holes; **c** Averaged stress evolution with zone variation in arteries in different reconstruction strategies. The stress values are calculated at the end of the loading step. **d** Comparison of the stress distribution among the reconstructed artery models with (d-1) SI/SP, (d-2) SI/RP, (d-3) 0.8-mm RI/RP, (d-4) 1.6-mm RI/RP, (d-5) 2.8-mm RI/RP, (d-6) 4.0-mm RI/RP and (d-7) 4.0-mm BRI/RP. The stress higher than the growth threshold, i.e., 0.3 MPa, is highlighted

Using the methods proposed in “Calculation of Stress Concentration Factor” Sect., the stress concentration factor *K* is calculated for all cases, as presented in Fig. 8a and b. The factor *K* in the model reconstructed with SI/RP is the highest among all cases. The stress concentration issue is significantly mitigated by introducing the punched hole. The application of a beveled hole can further decrease the value of the concentration factor *K* and reduce the number of stress concentration regions (Fig. 7d).

### Connection with Tissue Growth

The evolution of the averaged stress with variation of zones surrounding the incision vertex is plotted in Fig. 8c for different artery reconstruction strategies. A stress level of 0.3 MPa is adopted as the growth threshold [30], which is marked in Fig. 8c. According to the work by Holzapfel et al. [27] and Maltzahn et al. [31], the circumferential stress in the media, i.e., the smooth muscle layer, is significantly higher than that in the adventitia. We assume that the circumferential stress in the media dominates the growth threshold. Furthermore, the adventitia is assumed to be strongly bonded to the media. Therefore, the arterial wall was modeled as one layer, and the circumferential stress threshold in the media is adopted as the growth threshold of the arterial wall according to the work by Alford, et al. [30]. Arterial wall tissue growth is assumed to be obvious if the maximum principal stress is higher than this threshold. The stresses in all zones of the 4.0-mm RI/RP and 4.0-mm BRI/RP repaired arteries are lower than the growth threshold. For the 2.8-mm RI/RP and 1.6-mm RI/RP arteries, stress levels in most zones are higher than the threshold. The maximum principal stress in all cases at the end of the second loading step is compared in Fig. 8d, where the regions above the growth threshold are highlighted.

## Discussion

Sharp cracks and notches are natural sources of non-homogeneous stress distributions in materials [32–34]. Theoretically, these shapes can lead to stress singularity at the vertices of the crack and notch tips. Practically, the sharp geometrical shapes cause stress concentration issues in a variety of systems [35, 36]. In vascular surgery, an incision in arterial walls is one of those typical scenarios. A traditional incision includes a slit and two sharp vertex corners, located at both ends. To reconstruct the artery, the incision is filled with a patch, which adds materials to the original system. In this process, the incision is opened, and stress concentrates at the vertices. As a biological tissue, the arterial wall is sensitive to mechanical stimulation [6, 37]. Due to the geometrical singularity of the tips of incisions, the patch/incision connection introduces excess stress, which triggers abnormal tissue growth and remodeling. As a consequence, the arterial wall thickens and narrows—restenosis [10, 23, 38, 39], which can lead to non-lamellar flow and plaque reformation and undermine the success of endarterectomies. The present work proposes multiple arterial reconstruction strategies after femoral endarterectomies. Combining the surgery procedures and finite element simulations, the influence of different incision and patch geometries on the stress distribution and concentration in arterial walls is investigated and analyzed in detail.

The stress magnitude and distribution in reconstructed arterial walls are associated with the degree of match between the shapes of an incision and a patch. Particularly, the geometry of the incision and patch ends can significantly impact the strain of the arterial wall tissues. Currently, the prevalent incision in arteriotomy is a sharp-end incision, and the geometry of the commonly used patch in the subsequent angioplasty includes sharp-end and round-end shapes [5, 10, 23]. The comparison of the averaged stress and stress field distribution in reconstructed arteries with three different incision/patch combinations is shown in “Comparison Between Different Arteriotomy and Reconstruction Strategies” Sect. The averaged stress in the artery with an SI/RP strategy is the highest among the three scenarios (Fig. 5g). The stress concentration factor is also the largest (Fig. 8a and b). To fit the geometry of the patch, the arterial tissues around the incision corners are highly stretched (Fig. 5h), connoting the lack of compatibility between the geometry of the created incision and the applied patch. Although replacing the round-end patch with a sharp-end one can significantly decrease the stress level (Fig. 5g and h), the averaged stresses around the vertices are still higher than the stress threshold for tissue growth, i.e., 0.3 MPa [30]. These results are consistent with previous studies, which showcase high-level stress in the arterial tissues around the vertices of the sharp-end incision, reconstructed with a sharp-end patch [10], while the stress is more evenly distributed in the reconstructed artery using a round-end patch [5]. From the physical viewpoint, filling the incision in arterial walls, which is created during arteriotomy, with a patch is a procedure of material addition to the initial geometry. In the reconstructed artery, the angle subtended around the vertex of the incision rises above 360°, which is called the *excess cone* or *e-cone* [13, 14]. The presence of an *e-cone* is accompanied by stress concentration [15]. Moreover, it should be noted that the stress profiles in the arterial wall result from geometry and material mismatches.

According to the Neuber microsupport concept [40, 41], the sharp tip geometry of the incision can be defined as a notch based on the mechanics of continuum to mitigate the high stress and strain in the vicinity of the vertices. Motivated by this theory, the tissues at the vertices are removed by a vascular punch of 2.8 mm diameter to create holes after femoral endarterectomies, and a round-end patch is used in the angioplasty (Fig. 3c). The simulation results demonstrate the effectiveness of this approach. The averaged stress significantly decreases (Fig. 5c and f) and is the lowest among the three strategies, as illustrated in Fig. 5g. The high-stress zones are effectually spread out (Fig. 5h-3), and the stress concentration factor decreases to about 38% of that of the SI/RP case (Fig. 8a). From a clinical perspective, the use of a vascular punch allows for a cleaner arteriotomy in practice and is hypothesized to minimize endothelial denudation and disruption compared to a sharp technique using Potts scissors, which would yield smaller max in-plane principal stress. The smaller stress in the rounded notch configurations may also contribute to the reduction of inflammation from mechanical stresses, which can otherwise result in neointimal hyperplasia and stenosis as seen in stenting [42, 43].

While the stress in arterial walls declines due to the introduction of a 2.8-mm punched hole to the incision vertices, the averaged stress in Zone I to Zone VI is still above the tissue growth threshold, as shown in Fig. 5c and f, implying the arterial wall thickening and restenosis risk in these zones. To further reduce the risk of these issues and quantify the relationship between punch size and stress, another three punch sizes are considered (Fig. 4d–g). The comparison of magnitudes of stress in tissues across models with different sizes of punched holes reveals that the averaged stress drops in the scenario using a larger punch tip (Fig. 6g). The stress concentration factor $K$ shows a similar trend (Fig. 8b). However, this trend does not indicate that the optimal outcome is achieved with a larger punch tip size. When the punch size increases to 4.0 mm, the maximum averaged stresses are concurrently present in Zone I to III (Fig. 6c and f). This phenomenon is explained by the distribution of the stress field. Instead of only concentrating at the top point of the round-end incision as shown in the other three cases (Fig. 6h-1 to h-3), multiple high-level stress regions are observed (Fig. 7d-1), implying that introducing the 4.0-mm hole results in a larger stretch of the tissues in those zones. As a result, the included tissues could undergo thickening and lead to failure of femoral endarterectomies due to long-term restenosis. This issue can be effectively alleviated by simply modifying the standard hole to a beveled hole (Fig. 4h). The maximum averaged stress can be further reduced to around 86% of that in the model with a standard punched hole (Fig. 7c). Furthermore, the high-stress area and stress concentration factor $K$ are smaller in this case (Figs. 7d and 8c). Moreover, the artery repaired with 4.0-mm BRI/RP obviously has the most minor region subject to over-threshold stress and is at the lowest risk of tissue growth and restenosis among all considered scenarios (Fig. 8c and d).

There are several limitations associated with this study. First, blood flow-induced wall shear stress is not calculated in this study, which may underestimate the resultant stress. However, this work focuses on the influence of incision/patch geometry selection on the stress concentration in arterial walls. CFD simulations will be conducted in future work to better predict the resultant stress in the arterial wall. Second, the method used to evaluate stress concentration factor $K$, which could be more suitable for traditional engineering materials, is used to characterize the stress focusing level in arterial walls. This method may ignore the influence of the inelastic zone of nonlinear materials on the evaluation of $K$. An approach that is more applicable to nonlinear materials should be proposed for arterial wall tissues. Third, a rigid-patch assumption is made in this study by manipulating the boundary conditions of the incision. A generic modeling method will be proposed in future work to readily modify the mechanical properties of different patch materials. In addition, the arterial wall is built as a single-layer model. To more accurately predict arterial wall growth, a more detailed model involving different arterial layers and individual growth thresholds for these layers will be considered in the next work. A stress-driven growth and remodeling model will be developed and numerically implemented to predict the spatial and temporal change of arterial walls. Finally, arterial plaques, which stiffen the arterial wall, are removed from diseased arteries during the femoral endarterectomy. In this work, due to the lack of experimental data, the mechanical properties of postoperative arteries are assumed to restore to the level of the mechanical properties of near-normal arteries. Therefore, the Neo-Hookean model is applied to the postoperative arterial wall. A more accurate constitutive model will be developed to describe the mechanical behavior of postoperative arteries when the relevant experimental data become available.

Restenosis is a long-term complication of endarterectomies, which is the result of arterial wall thickening induced by excessive stress stimulation. In the present work, the impact of different arteriotomy and angioplasty strategies on the mechanical response of arterial wall tissues is systematically studied by combining realistic surgical procedures and finite element simulations. Based on the surgical and simulation results, the main conclusions of the present work are summarized as follows:

- Three types of incision/patch pairs with different geometries are simulated according to femoral endarterectomies. The simulation results obtained from the reconstruction regimes with SI/SP and SI/RP are qualitatively consistent with the previous data, which validates the proposed computational methods.
- Punching the vertices of the sharp-end incision with a vascular punch to create a round-end incision is an effective method to decrease the stress magnitude and mitigate stress concentration in the region around the incision vertices. The method shows great potential to alleviate detrimental arterial remodeling and hyperplasia and promote the long-term outcome of femoral endarterectomies.
- Increasing the size of the punched hole significantly minimizes the stress level and spreads the high-stress region to a larger zone. With the punch size approaching the diameter of the round patch ends, the high-stress region splits and distributes surrounding the punched hole. Modifying the standard circular hole to a beveled hole can effectively mitigate the issue and further reduce the stress level and concentration factor.
- The averaged stress around the incision ends in the arterial walls repaired with the 4.0-mm BRI/RP and 4.0-mm RI/RP methods is below the growth threshold, which implies a low risk of restenosis.

## Figures and Tables

**Fig. 9:**
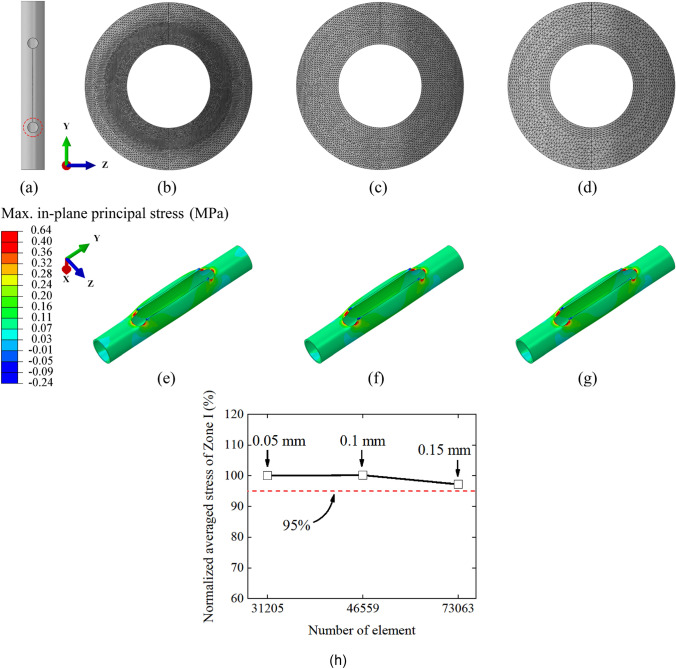
Mesh convergence study: **a** The computational model of the artery, reconstructed with a 4.0-mm RI/RP; The mesh of the region as marked in a (red dashed circle) with an element size of **b** 0.05 mm, **c** 0.1 mm, and **d** 0.15 mm; Comparison of the stress distribution and magnitude among the computational models with element sizes of **e** 0.05 mm, **f** 0.1 mm, and **g** 0.15 mm in the region as shown in b, c, and d, respectively; **h** The averaged stress of Zone I in the models with different element sizes and numbers of elements. The stress is normalized by the stress (0.246 MPa) in the model with an element size of 0.05 mm

## Appendix A: Mesh Convergence Study

The computational model of the artery, reconstructed with a 4.0-mm RI/RP, is used as an example to demonstrate the mesh convergence of the simulations in this work. Three mesh sizes, i.e., 0.05, 0.1, and 0.15 mm, as shown in Fig. 9b–d, are utilized to discretize the region within Zone VI surrounding the punch holes (see Fig. 9a). The stress distribution and magnitude are demonstrated in Fig. 9e–g. No significant difference between the stress distribution and magnitude is observed according to the results. The stress difference in the models with different element sizes and numbers of elements is less than 5%, as presented in Fig. 9h. The results indicate the convergence of the used element size.

## Declarations

### Clinical Trial Registration

This study is not a clinical trial. Clinical trial number: not applicable.
